# Impact of ventricular tachycardia ablation in subcutaneous implantable cardioverter defibrillator carriers: a multicentre, international analysis from the iSUSI project

**DOI:** 10.1093/europace/euae066

**Published:** 2024-04-08

**Authors:** Marco Schiavone, Alessio Gasperetti, Paolo Compagnucci, Julia Vogler, Mikael Laredo, Elisabetta Montemerlo, Simone Gulletta, Alexander Breitenstein, Matteo Ziacchi, Martin Martinek, Michela Casella, Pietro Palmisano, Lukas Kaiser, Carlo Lavalle, Leonardo Calò, Sebastian Seidl, Ardan M Saguner, Giovanni Rovaris, Jürgen Kuschyk, Mauro Biffi, Luigi Di Biase, Antonio Dello Russo, Claudio Tondo, Paolo Della Bella, Roland Tilz, Giovanni B Forleo, iSUSI investigators, M Schiavone, A Gasperetti, R Arosio, M Viecca, G B Forleo, M Ziacchi, I Diemberger, A Angeletti, M Biffi, N Fierro, S Gulletta, P Della Bella, C Tondo, G Mitacchione, A Curnis, P Compagnucci, M Casella, A Dello Russo, L Santini, C Pignalberi, M Magnocavallo, A Piro, C Lavalle, F Picarelli, D Ricciardi, E Bressi, L Calò, E Montemerlo, G Rovaris, S De Bonis, A Bisignani, G Bisignani, G Russo, E Pisanò, P Palmisano, F Guarracini, F Vitali, M Bertini, J Vogler, T Fink, R Tilz, F Fastenrath, J Kuschyk, L Kaiser, S Hakmi, M Laredo, X Waintraub, E Gandjbakhch, N Badenco, A Breitenstein, A M Saguner, M Martine, S Seidl, L Di Biase

**Affiliations:** Department of Clinical Electrophysiology & Cardiac Pacing, Centro Cardiologico Monzino, IRCCS, Milan, Italy; Department of Systems Medicine, University of Rome Tor Vergata, Rome, Italy; Cardiology Unit, Luigi Sacco University Hospital, Via Giovanni Battista Grassi, 74, Milan 20157, Italy; Department of Cardiology, Johns Hopkins University, 1800 Orleans Street, Baltimore, MD 21218, USA; Cardiology and Arrhythmology Clinic, University Hospital ‘Ospedali Riuniti’, Ancona, Italy; Department of Rhythmology, University Heart Center Lübeck, Lubeck, Germany; Institut de Cardiologie, Groupe Hospitalier Pitié-Salpêtrière and Sorbonne Université, Paris, France; Cardiology Unit, ASST Monza, San Gerardo Hospital, Monza, Italy; Arrhythmology and Electrophysiology Unit, San Raffaele Hospital, IRCCS, Milan, Italy; Cardiology Clinic, University Hospital Zurich, Zurich, Switzerland; Cardiology Unit, IRCCS, Department of Experimental, Diagnostic and Specialty Medicine, Sant'Orsola Hospital, University of Bologna, Bologna, Italy; Internal Medicine 2 with Cardiology, Angiology, and Intensive Care Medicine, Ordensklinikum Linz Elisabethinen, Linz, Austria; Cardiology and Arrhythmology Clinic, University Hospital ‘Ospedali Riuniti’, Ancona, Italy; Cardiology Unit, ‘Card. G. Panico’ Hospital, Tricase, Italy; Department of Cardiology and Critical Care Medicine, St. George Klinik Asklepios, Hamburg, Germany; Department of Clinical, Internal, Anesthesiology and Cardiovascular Sciences, Sapienza University of Rome, Rome, Italy; Department of Cardiology, Policlinico Casilino, Rome, Italy; Internal Medicine 2 with Cardiology, Angiology, and Intensive Care Medicine, Ordensklinikum Linz Elisabethinen, Linz, Austria; Cardiology Clinic, University Hospital Zurich, Zurich, Switzerland; Cardiology Unit, ASST Monza, San Gerardo Hospital, Monza, Italy; Cardiology Unit, University Medical Centre Mannheim, Manheim, Germany; Cardiology Unit, IRCCS, Department of Experimental, Diagnostic and Specialty Medicine, Sant'Orsola Hospital, University of Bologna, Bologna, Italy; Cardiac Arrhythmia Center, Division of Cardiology, Montefiore-Einstein Center, Bronx, NY, USA; Cardiology and Arrhythmology Clinic, University Hospital ‘Ospedali Riuniti’, Ancona, Italy; Department of Clinical Electrophysiology & Cardiac Pacing, Centro Cardiologico Monzino, IRCCS, Milan, Italy; Department of Biomedical, Surgical and Dental Sciences, University of Milan, Milan, Italy; Arrhythmology and Electrophysiology Unit, San Raffaele Hospital, IRCCS, Milan, Italy; Department of Rhythmology, University Heart Center Lübeck, Lubeck, Germany; Cardiology Unit, Luigi Sacco University Hospital, Via Giovanni Battista Grassi, 74, Milan 20157, Italy

**Keywords:** Subcutaneous ICD, Ventricular tachycardia, Catheter ablation, Defibrillator, Arrhythmic mortality

## Abstract

**Aims:**

Catheter ablation (CA) of ventricular tachycardia (VT) has become an important tool to improve clinical outcomes in patients with appropriate transvenous implantable cardioverter defibrillator (ICD) shocks. The aim of our analysis was to test whether VT ablation (VTA) impacts long-term clinical outcomes even in subcutaneous ICD (S-ICD) carriers.

**Methods and results:**

International Subcutaneous Implantable Cardioverter Defibrillator (iSUSI) registry patients who experienced either an ICD shock or a hospitalization for monomorphic VT were included in this analysis. Based on an eventual VTA after the index event, patients were divided into VTA+ vs. VTA− cohorts. Primary outcome of the study was the occurrence of a combination of device-related appropriate shocks, monomorphic VTs, and cardiovascular mortality. Secondary outcomes were addressed individually. Among *n* = 1661 iSUSI patients, *n* = 211 were included: *n* = 177 experiencing ICD shocks and *n* = 34 hospitalized for VT. No significant differences in baseline characteristics were observed. Both the crude and the yearly event rate of the primary outcome (5/59 and 3.8% yearly event rate VTA+ vs. 41/152 and 16.4% yearly event rate in the VTA−; log-rank: *P* value = 0.0013) and the cardiovascular mortality (1/59 and 0.7% yearly event rate VTA+ vs. 13/152 and 4.7% yearly event rate VTA−; log-rank *P* = 0.043) were significantly lower in the VTA + cohort. At multivariate analysis, VTA was the only variable remaining associated with a lower incidence of the primary outcome [adjusted hazard ratio 0.262 (0.100–0.681), *P* = 0.006].

**Conclusion:**

In a real-world registry of S-ICD carriers, the combined study endpoint of arrhythmic events and cardiovascular mortality was lower in the patient cohort undergoing VTA at long-term follow-up.

**ClinicalTrials.gov identifier:**

NCT0473876.

## Graphical Abstract

**Graphical Abstract:**
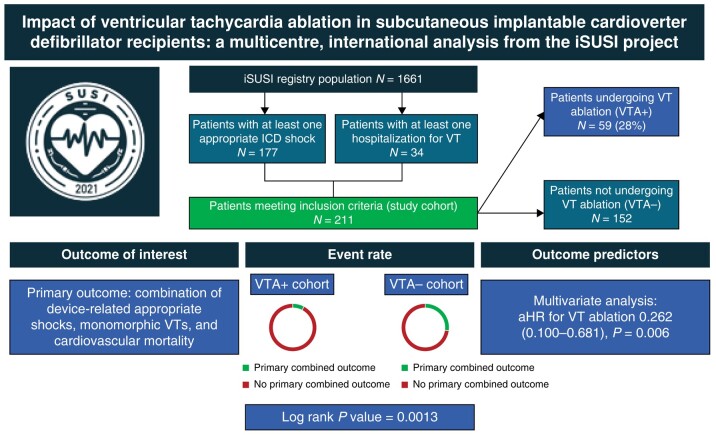

**What’s new?**

- Among all the patients enrolled in the International Subcutaneous Implantable Cardioverter Defibrillator (iSUSI) registry, 211 patients experienced either appropriate shock or a hospitalization due to monomorphic ventricular tachycardia (VT) episodes. After the index event, *n* = 59 (28.0%) patients underwent VT ablation (VTA) (VTA+ cohort).
- Over 17.3 (10.4–34.8) months from the index event, patients in the VTA+ cohort experienced both significantly lower primary combined event rates (5/59 vs. 41/152, log-rank *P* value = 0.0013) and lower cardiovascular mortality (1/59 vs. 13/152; log-rank *P* value = 0.0432).
- Among all tested characteristics, VTA was the only variable remaining significantly associated with a lower rate of primary combined event rates at multivariate analysis [adjusted hazard ratio 0.262 (0.100–0.681), *P* = 0.006].

## Introduction

The most recent European Guidelines on ventricular arrhythmia (VA) management recommend ventricular tachycardia (VT) ablation (VTA) in patients with structural heart disease and recurrent VT episodes resulting in implantable cardioverter defibrillator (ICD) appropriate shocks.^1^ A high incidence of appropriate ICD shocks, in fact, has been associated with a negative effect on quality of life and survival in ICD carriers.^2,3^ While medical treatment of VAs could potentially reduce the overall VA burden and the subsequent ICD shocks, in patients with structural heart diseases, medical management is oftentimes limited to the use of beta-blockers and amiodarone, with both medications hampered by side effects and long-term unsatisfactory efficacy.^4^ Thanks to definite progress that has been made in this field in the recent years,^5^ catheter ablation (CA) has become an increasingly more important tool to avoid VT recurrences and limit ICD therapies, thereby potentially improving clinical outcomes, even over antiarrhythmic drug therapy in specific settings.^6–8^ However, randomized control trials addressing the outcomes of CA at the time of transvenous (TV) ICD implantation have shown conflicting results when assessing the reduction of VT burden or mortality.^9–11^ Regardless of the VTA timing, data regarding the generalizability of VTA trials’ findings in subcutaneous ICD (S-ICD) carriers are currently lacking. Thus, a reduction of VT burden in S-ICD carriers, potentially resulting in significantly lower appropriate shock rates due to the lack of antitachycardia pacing (ATP) in these devices,^12^ has never been investigated so far. Moreover, these patients often represent a clinically different entity, as the S-ICD device is often used in real-world practice in young patients with inherited heart disease or in patients with high-infective risk and/or previous TV-ICD failures.^13,14^ Therefore, the objective of this analysis, based on the International Subcutaneous Implantable Cardioverter Defibrillator (iSUSI) registry, was to assess whether VTA following an ICD shock or hospitalization for monomorphic VT has an impact on the long-term clinical outcomes of S-ICD carriers.

## Methods

The iSUSI—former ELISIR project—is a multi-centre, open-label, independent, and physician-initiated observational registry, whose characteristics have been previously described.^15^ At the time of this manuscript drafting, a total of 24 public and private healthcare institutions from 6 different countries in Europe and in the USA were involved in the registry. All consecutive patients meeting current guideline indications for ICD implantation and undergoing implantation of an S-ICD device (Boston Scientific, Marlborough, MA, USA) at one of the participating institutions were enrolled in our registry. This manuscript has been drafted in accordance with the tenets of the Helsinki Declaration and has been approved by the local institutional review board. Data supporting this study are available upon reasonable request to the corresponding author.

### Data collection

Data collection methods for the patients enrolled in this registry have been previously presented.^16^ In brief, for each enrolled patient, baseline and procedural characteristics were collected in accordance with a centralized spreadsheet, clearly defining each research item. At the time of S-ICD placement, details regarding device positioning and incision technique were retrieved. The PRAETORIAN score was collected as per existing definition, either from a two-projection post-procedural chest X-ray or intra-procedurally.^17–19^ Baseline device programming setup and the rate of SMART Pass algorithm use for inappropriate shock risk reduction^20^ were collected as well. Follow-up strategy was left to each centre’s policy, with most patients being evaluated at 1, 6, and 12 months and every 6 months thereafter. All device therapy delivered over the entirety of follow-up (appropriate, inappropriate, and ineffective) and/or arrhythmia recorded during in-hospital and/or remote follow-up and/or in-clinic device interrogation were collected, as well as in-hospital admissions (such as for VA events or VTA procedures), cardiovascular, and overall mortality.

### Aim of the study, cohort, and outcome definition

All patients enrolled in the iSUSI registry that experienced the combined index event of either ICD shock or hospitalization for monomorphic VT at any time during clinical follow-up were extracted and included in the study. Based on an eventual VTA after the index event, enrolled patients were divided into the two study cohorts:

- Patients who were treated with a VTA (VTA+ cohort)
- Patients who were not treated with a VTA (VTA− cohort)

Time zero was set as time of index event occurrences. In case of patients experiencing both different index events, time zero was set as the time of first index event occurrences.

**Figure 1:**
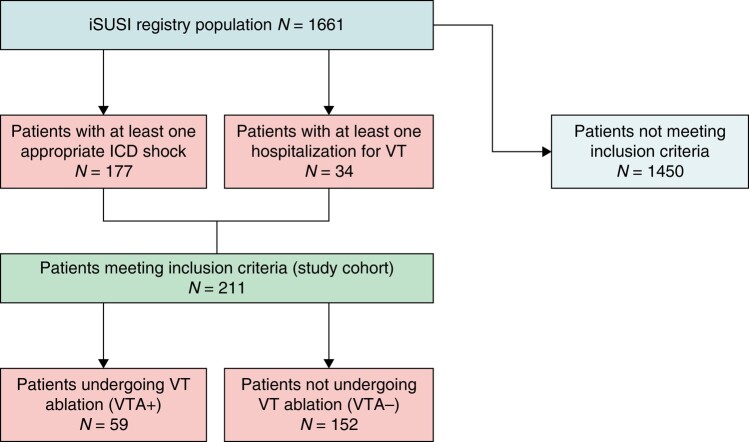
Flowchart summarizing the workflow resulting in the two final cohorts.

The primary outcome of the study was defined as the occurrence of a combination of device-related appropriate shocks, monomorphic VTs, and cardiovascular mortality during follow-up after index event. Outcome occurrence has been expressed both as absolute number and as rate calculated as event/patient-years. Secondary outcomes were device-related appropriate shocks, monomorphic VTs, and cardiovascular mortality, each one addressed individually. *Figure 1* displays the flowchart summarizing the workflow resulting in the two final cohorts.

### Statistical analysis

Normality of distribution of continuous numerical variables was tested using the Shapiro–Wilk test for normality. Continuous variables were reported as mean ± standard deviation (SD) or as median [inter-quartile range (first to third quartiles) (IQR)] if normally or non-normally distributed, respectively. Categorical variables were reported as count (%). Comparisons have been performed using a χ^2^ test or a Fisher’s exact test between categorical variables. For comparisons between numerical variables, the independent Student’s *t*-test or a Mann–Whitney *U* test (or their paired equivalents for paired analysis) was used, as appropriate according to their distribution. Event-free survival was plotted using Kaplan–Meier estimates, and a log-rank test was used to compare differences in survivals. A Cox regression was used to assess the associations between baseline and procedural characteristics and the primary outcome. Time of censoring was set either as the time of the outcome or the time of last follow-up, whichever came first. Univariable analyses were performed at first, reporting unadjusted hazard ratios (HR); all variables reaching a threshold *P* value 0.05 were then fit into a multivariable model to adjust for confounders, from which adjusted hazard ratios (aHR) were retrieved. An additional Cox regression was performed, using cardiovascular (CV) mortality as clinical endpoint. Due to the relatively low event rate, univariate analysis only has been performed to avoid data overfitting. A two-sided *P* value < 0.05 was considered significant throughout the manuscript. All analyses were performed using Stata 14.0 (StataCorp LLC, College Station, TX).

## Results

### Patient population

Among the 1661 patients enrolled in the iSUSI registry, a total of 211 patients were included in the current study. After the index event, *n* = 59 (28.0%) underwent VTA (VTA+ cohort). Alongside, we found *n* = 23 sustained VT events not treated by an appropriate S-ICD intervention (thereby not falling in the device shock zone) and not resulting in patients’ hospitalization. As shown in *Table 1*, no significant differences in baseline characteristics at study enrolment were observed between the two study cohorts including age and gender distribution (47.7 ± 16.1 years VTA+ vs. 47.9 ± 17.0 years VTA−, *P* = 0.946; 83.1% vs. 82.4%, *P* = 0.889) as well as cardiovascular comorbidities. The only exception was the presence of diabetes (5.1% vs. 18.5%, *P* = 0.015). The most common underlying cardiac substrate were ischaemic and dilatative cardiomyopathies, in both cohorts (32.2% vs. 32.2%, *P* = 0.996, and 15.3% vs. 18.4%, *P* = 0.587, respectively). Subcutaneous implantable cardioverter defibrillator implanting techniques and device programming settings resulted uniform between the two patient populations.

**Table 1:** Baseline characteristics

	VTA+ (*n* = 59)	VTA− (*n* = 152)	*P*
Age (years), mean ± SD	47.7 ± 16.1	47.9 ± 17.0	0.946
Male, *n* (%)	49 (83.1)	125 (82.4)	0.889
LVEF (%), mean ± SD	43.8 ± 13.2	42.3 ± 15.5	0.517
Primary prevention implant, *n* (%)	21 (35.6)	66 (43.7)	0.283
Hypertension, *n* (%)	22 (37.3)	51 (39.2)	0.799
Diabetes, *n* (%)	3 (5.1)	24 (18.5)	**0**.**015**
CKD, *n* (%)	5 (8.5)	21 (16.2)	0.156
Substrate			
Ischaemic cardiomyopathy, *n* (%)	19 (32.2)	49 (32.2)	0.996
DCM, *n* (%)	9 (15.3)	28 (18.4)	0.587
HCM, *n* (%)	3 (5.1)	11 (7.2)	0.573
Brugada, *n* (%)	2 (3.4)	9 (5.9)	0.458
ARVC, *n* (%)	6 (10.2)	11 (7.2)	0.482
Idiopathic VF, *n* (%)	8 (13.6)	19 (12.5)	0.836
Valvular cardiomyopathy, *n* (%)	0	2 (1.3)	0.376
LQTS, *n* (%)	0	6 (3.9)	0.121
Myocarditis, *n* (%)	4 (6.8)	6 (4.0)	0.385
Other, *n* (%)	8 (13.6)	11 (7.2)	0.150
S-ICD placement details			
Two-incision technique, *n* (%)	54 (91.5)	139 (92.1)	0.900
Inter-muscular placement, *n* (%)	43 (72.9)	118 (78.2)	0.418
PRAETORIAN score available, *n* (%)	51 (86.4)	122 (80.2)	0.296
Low risk (<90)^a^, *n* (%)	47 (92.1)	116 (95.1)	0.452
S-ICD programming details			
Conditional zone (b.p.m.), median (IQR)	200 (200–220)	200 (190–220)	0.385
Shock zone (b.p.m.), median (IQR)	240 (230–250)	240 (230–250)	0.189
Vector programming			
Primary, *n* (%)	38 (64.4)	92 (60.5)	0.603
Secondary, *n* (%)	14 (23.7)	44 (28.9)	0.446
Alternative, *n* (%)	7 (11.8)	16 (10.5)	0.780
SMART Pass algorithm, *n* (%)	51 (86.4)	120 (78.9)	0.212
Medical treatment			
Beta-blockers, *n* (%)	44 (74.6)	99 (65.1)	0.187
IC, *n* (%)	2 (3.4)	2 (1.3)	0.321
Amiodarone, *n* (%)	14 (20.4)	31 (23.7)	0.596
Mexiletine, *n* (%)	2 (3.4)	1 (0.7)	0.132
Sotalol, *n* (%)	3 (5.1)	6 (3.9)	0.714
Patients experiencing at study inclusion			
Appropriate shock(s), *n* (%)	48 (81.3)	130 (85.5)	0.454
Num. of appropriate shocks, median (IQR)	2 (1–4)	2 (1–3)	0.590
Hospitalizations for monomorphic VT(s), *n* (%)	11 (18.7)	23 (15.1)	0.533

Statistically significant *P* values have been boldened.

ARVC, arrhythmogenic right ventricular cardiomyopathy; CKD, chronic kidney disease; DCM, dilated cardiomyopathy; HCM, hypertrophic cardiomyopathy; IC, class Ic anti-arrhythmic drugs; IQR, inter-quartile range; LVEF, left ventricular ejection fraction; LQTS, long-QT syndrome; SD, standard deviation; S-ICD, subcutaneous implantable cardioverter defibrillator; VT, ventricular tachycardia; VF, ventricular fibrillation.

^a^Percentages are calculated on the total of patients for which a PRAETORIAN Score is available.

The index event leading to study inclusion was similar between the two cohorts; receiving an appropriate shock was the most common index event leading to study inclusion (*n* = 178; 81.3% VTA+ vs. 85.5% VTA−, *P* = 0.454). At the time of the index event, the median number of appropriate S-ICD shocks was *n* = 2 (1–4) (VTA+) vs. *n* = 2 (1–3) (VTA−), *P* = 0.590.

### Ventricular tachycardia ablation in patients with subcutaneous implantable cardioverter defibrillator

**Figure 2:**
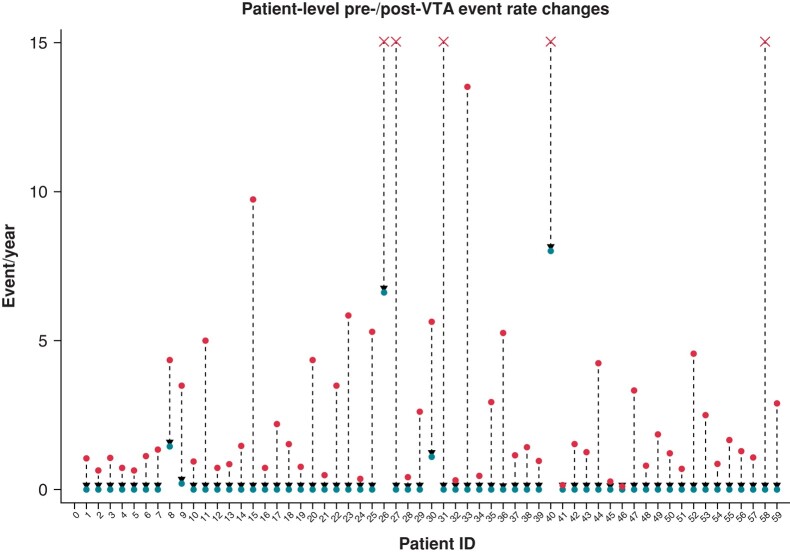
Changes in pre- (upper end)/post- (lower end) ventricular tachycardia (VT) event rates at individual patient level. ‘X’ indicates a pre-ventricular tachycardia ablation (VTA) rate capping at 15 for graphical reasons.

A total of 59 patients underwent VTA within 4 (2–7) months after index event. The exact rate of patients undergoing endocardial-only VTA vs. endo-epicardial approach was 78% (*n* = 46) vs. 22% (*n* = 13); the most common VTA strategy was substrate-based approach,^21,22^ employed as the first-line strategy in *n* = 36 (61%) of cases. Complete acute procedural success was achieved in *n* = 52 (88%) of cases, while in *n* = 7 (12%) of cases, a partial success was obtained. No major peri-procedural complications were reported. Over a median follow-up of 23.7 (14.2–40.4) months from VTA, 5/59 patients experienced the primary outcome (*n* = 4 appropriate shocks; *n* = 1 monomorphic VTs; *n* = 1 CV death). Comparing individual rates of arrhythmic events pre- and post-VTA, a significant decrease in these events was observed [median events pre-VTA 2 (2–4) vs. median events post-VTA 0 (0–0), paired *P* < 0.001; event rate pre-VTA 1.4 event/year vs. event rate post-VTA 0.1 event/year, paired *P* < 0.001]. *Figure 2* graphically shows event rate reduction at an individual patient level.

### Cohort comparisons and outcome predictors

**Figure 3:**
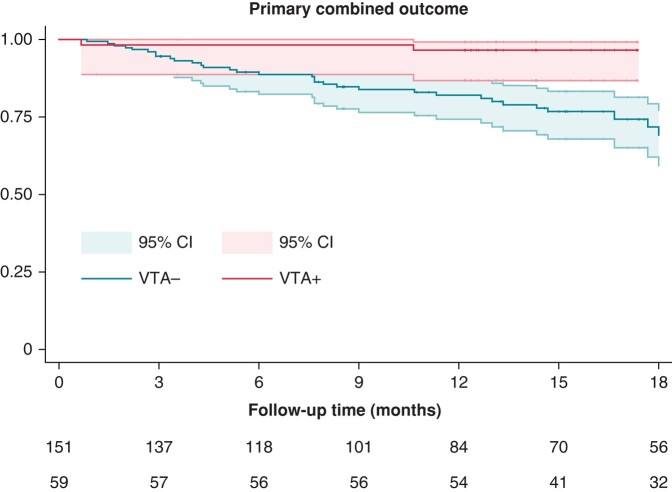
Kaplan–Meier curves for the primary outcome of interest. Red: patients undergoing ventricular tachycardia ablation (VTA+ cohort). Blue: patients not undergoing ventricular tachycardia ablation (VTA− cohort).

Overall, the median follow-up of the study was 17.3 (10.4–34.8) months, and the combined primary outcome was experienced by 46 (21.8%) patients. Even if a slightly longer follow-up time was observed in in the VTA+ cohort [23.7 (14.2–40.4) vs. 15.4 (8.4–33.0), *P* = 0.004], both the crude and the yearly event rate of the primary outcome (5/59 and 3.8% yearly event rate VTA+ vs. 41/152 and 16.4% yearly event rate in the VTA−; log-rank *P* value = 0.0013) and the cardiovascular mortality (1/59 and 0.7% yearly event rate VTA+ vs. 13/152 and 4.7% yearly event rate VTA−, log-rank *P* = 0.043) were significantly lower in the VTA+ cohort. *Figure 3* displays Kaplan–Meier curves for the two cohorts with the primary outcome of interest. *Table 2* reports follow-up data for both patient cohorts.

**Table 2:** Follow-up data

	VTA+ (*n* = 59)	VTA− (*n* = 152)	*P*
Follow-up time (months), median (IQR)	23.7 (14.2–40.4)	15.4 (8.4–33.0)	**0**.**004**
Patients experiencing combined outcome, *n* (%)	5 (8.5)	41 (27.0)	**0**.**004**
ICD appropriate shock, *n* (%)	4 (6.7)	26 (17.1)	0.053
Monomorphic VT, *n* (%)	1 (1.7)	9 (5.9)	0.194
Cardiovascular death, *n* (%)	1 (1.7)	13 (8.6)	0.073
Inappropriate shocks, *n* (%)	3 (5.1)	12 (7.9)	0.478

Statistically significant *P* values have been boldened.

ICD, implantable cardioverter defibrillator; IQR, inter-quartile range; VT, ventricular tachycardia.

At multivariate Cox regression, VTA was associated with a lower incidence of the primary outcome [aHR 0.262 (0.100–0.681), *P* = 0.006], even after accounting for all other variables that were significant at univariate [i.e. left ventricular ejection fraction (LVEF): HR/% increase 0.972 (0.953–0.993), *P* = 0.009; diabetes: HR 2.985 (1.086–4.863), *P* = 0.030; chronic kidney disease (CKD): HR 2.173 (1.058–4.464), *P* = 0.034; ischaemic cardiomyopathy (CMP): HR: 2.332 (1.288–4.223), *P* = 0.005]. *Table 3* reports the final multivariate model. *Table 4* reports the univariate Cox regression using only CV mortality as an endpoint.

**Table 3:** Primary outcome predictors

Predictor	HR	CI	*P*	aHR	CI	*P*
Age	1.005	(0.986–1.023)	0.606			
Sex	1.018	(0.473–2.193)	0.963			
LVEF	0.972	(0.953–0.993)	**0.009**	0.990	(0.965–1.014)	0.414
Primary prevention implant	1.658	(0.921–2.986)	0.092			
Hypertension	1.140	(0.602–2.158)	0.688			
Diabetes	2.985	(1.086–4.863)	**0**.**030**	1.322	(0.603–2.897)	0.485
CKD	2.173	(1.058–4.464)	**0**.**034**	1.304	(0.608–2.795)	0.495
Ischaemic cardiomyopathy	2.332	(1.288–4.223)	**0**.**005**	1.781	(0.875–3.628)	0.112
Two-incision technique	1.553	(0.480–5.023)	0.462			
Inter-muscular device	1.261	(0.633–2.511)	0.509			
VT ablation	0.215	(0.100–0.637)	**0**.**004**	0.262	(0.100–0.681)	**0**.**006**

Statistically significant *P* values have been boldened.

aHR, adjusted hazard ratio; CI, confidence interval; CKD, chronic kidney disease; CMP, cardiomyopathy; HR, hazard ratio; LVEF, left ventricular ejection fraction; VT, ventricular tachycardia.

**Table 4:** Cardiovascular mortality outcome predictors

Predictor	HR	CI	*P*
Age	1.037	(1.001–1.007)	**0**.**043**
Sex	0.587	(0.181–1.875)	0.365
LVEF	0.924	(0.882–0.967)	**0**.**001**
Primary prevention implant	1.918	(0.659–5.583)	0.232
Hypertension	2.458	(0.694–8.717)	0.164
Diabetes	1.704	(0.360–8.044)	0.501
CKD	4.109	(1.158–14.470)	**0**.**029**
Ischaemic cardiomyopathy	3.979	(1.296–12.214)	**0**.**016**
Two-incision technique	1.342	(0.174–10.361)	0.778
Inter-muscular device	1.173	(0.359–3.825)	0.791
VT ablation	0.159	(0.021–1.223)	**0**.**077**

Statistically significant *P* values have been boldened.

CI, confidence interval; CKD, chronic kidney disease; HR, hazard ratio; LVEF, left ventricular ejection fraction; VT, ventricular tachycardia.

## Discussion

This analysis is the first, large, multicentred, cohort study assessing the outcomes of VTA in S-ICD carriers. The main results of our analysis can be summarized as follows:

- Among all the patients enrolled in the iSUSI registry, 211 patients experienced the index event of either appropriate shock or a hospitalization due to monomorphic VT episodes. After the index event, *n* = 59 (28.0%) patients underwent VTA (VTA+ cohort), on average within 4 (2–7) months.
- Over a median follow-up of 17.3 (10.4–34.8) months from the index event, the combined primary outcome of device-related appropriate shocks, monomorphic VTs, and cardiovascular mortality was experienced by 46 (21.8%) patients in the overall cohort.
- Patients in the VTA+ cohort experienced both significantly lower primary combined event rates (5/59 vs. 41/152, log-rank *P* value = 0.0013) and lower cardiovascular mortality (1/59 vs. 13/152; log-rank *P* value = 0.0432).
- Among all tested characteristics, VTA was the only variable remaining significantly associated with a lower rate of primary combined event rates at multivariate analysis [aHR 0.262 (0.100–0.681), *P* = 0.006].

### Ventricular tachycardia burden reduction and cardiovascular outcomes

Previous randomized clinical trials have mostly assessed the role of prophylactic CA, without providing definite results in terms of cardiovascular mortality reduction. The Substrate Mapping and Ablation in Sinus Rhythm to Halt Ventricular Tachycardia (SMASH-VT) postulated that a decreased rate of VT recurrences may be linked to a clinical benefit in this regard.^9^ On the other hand, the Substrate Modification in Stable Ventricular Tachycardia in Addition to Implantable Cardioverter Defibrillator Therapy (VTACH)^23^ and the Substrate Modification Study (SMS)^24^ trials failed to prove a survival benefit, probably due the higher rate of VT episodes in both arms alongside with different clinical characteristics. The most recent PARTITA trial^11^ addressed instead the role of VTA in patients experiencing appropriate ICD shocks, constituting a ‘secondary prevention’ approach. This trial demonstrated the benefits of VTA by reducing the occurrences of both deaths and heart failure (HF) episodes in the VTA group. However, it is crucial to note that the Phase B hard endpoint of this trial was a composite of death from any cause or worsening HF leading to hospitalization. Notably, the focus was not exclusively on cardiovascular deaths, a significant consideration given the overall limited number of events. To summarize this evidence, a meta-analysis from Prasitlumkum *et al.*^25^ recently evaluated nine randomized controlled trials (*n* = 1106 TV-ICD patients) analysing the impact of early VTA in patients with an ICD. The authors concluded that early CA was beneficial in reducing VT burden and ICD therapies, although not affecting mortality rate and quality of life of TV-ICD patients.

All trials published so far analysing the benefit of VTA in ICD carriers, however, have enrolled TV-ICD patients, who are known to represent a completely different population for their clinical baseline characteristics when compared with S-ICD patients. Especially in a non-trial clinical setting, in fact, most studies have reported S-ICD carriers to be either younger, active individuals affected by inherited heart diseases or elderly patients with multiple comorbidities and risk factors for traditional TV-ICD.^26–29^ Moreover, the lack of ATP in S-ICD devices may potentially expose S-ICD carriers both to a higher number of shocks due to high ventricular rate monomorphic VTs and to prolonged monomorphic VTs. The relative impact of ICD shock reduction on long-term clinical outcome in S-ICD carriers cannot therefore be directly translated from the currently available evidence. This study tries to partially fill that evidence void, reporting the clinical outcome of S-ICD patients undergoing VTA after significant VA events. At an individual patient level, VTA was demonstrated to be associated with a very significant event rate reduction.^25^ In our cohort, this reduction was associated with a significant improvement of the considered clinical outcomes in patients undergoing VTA, potentially supporting a significant clinical role for this procedure in this setting.

However, some differences between the analysis conducted on this registry and the population previously enrolled in the other TV-ICD based trials must be acknowledged. First, our inclusion criteria allowed for study inclusion also upon a hospitalization for monomorphic VT. While those events were clearly clinically meaningful, due to the necessity of a contextual patient hospitalization, there is no evidence that those events would not have been terminated by ATP, thereby not leading to patient inclusion in other trials enrolling TV-ICD patients. Nevertheless, it should be highlighted that the potential need for ATP is often difficult to define *a priori.*^30^ Patients with a history of monomorphic VTs were likely to be implanted with a TV-ICD in the first place and thereby were less likely to be included in the iSUSI project. An exception in this group might be represented by patients with a very high infective risk or absolute contraindications to TV devices, but the accurate rate is arduous to estimate. Moreover, apart from the potential risks of accelerating VTs, the ATP success rate for terminating VTs ranges from 62% to 84%,^31,32^ with the lowest rates being observed in the fast VT zone programming, with these arrhythmias being more likely to result in acute HF and hospitalizations, as in our S-ICD cohort. Notwithstanding, this potentially slightly distorts the characteristics of our cohort compared with the patients enrolled in TV-ICD trials. This inclusion criterion, however, was set as those monomorphic VT episodes representing important clinical episodes for S-ICD carriers. Additionally, <20% of the whole cohort has been enrolled upon this criterion.

Finally, while VTA was associated with a clinical arrhythmic benefit in our cohort, we did not observe a formal statistical significance for individual CV mortality reduction with VTA [HR: 0.159 (0.021–1.223); *P* = 0.077]. These findings are in line with what was reported by Prasitlumkum *et al.*,^25^ demonstrating that early VTA in TV-ICD patients was not associated with all-cause mortality [pooled odds ratio (OR) 0.91, 95% CI 0.63–1.31 with *I*^2^ = 0%; *P* = 0.6] and CV mortality rate (pooled OR 0.82; 95% CI 0.51–1.32 with *I*^2^ = 0%; *P* = 0.41) benefits while being correlated with reduced VT recurrences (pooled OR 0.64; 95% CI 0.46–0.87 with *I*^2^ = 19.6%; *P* = 0.007) and ICD shocks (pooled OR 0.53; 95% CI 0.35–0.79 with *I*^2^ = 45.5%; *P* = 0.002).

### Clinical implications

Routine prophylactic CA for VT events has failed to provide a clinical net benefit in multiple large trials,^23,24^ with the BERLIN VT trial being interrupted for futility representing the final nail in the coffin for this approach.^10^ The main argument against prophylactic VTA is the difficulty in routinely proving a net benefit gain in a patient population that may not have VA recurrences in the future, in front of the immediate cost and the potential peri-procedural complications of an invasive complex procedure. This problem may ultimately be a matter of risk stratification and patient selection. On the other hand, the recent PARTITA trial identified a very adequate patient population that could benefit from VTA while providing new evidence that early VTA significantly improves hard endpoints in TV-ICD carriers experiencing appropriate shocks. Indeed, the study was limited by the small number of randomized patients and by the relatively small number of CAs that prevented the authors to draw definite conclusions regarding the safety of this invasive strategy. Therefore, while additional randomized controlled studies will be required to fully capture the effect size of VTA in S-ICD carriers, the observational evidence from our registry seem to provide clinical rationale to the extension of a similar approach even to patients implanted with S-ICD experiencing appropriate shocks or clinically significant monomorphic VT events.

### Limitations and pitfalls

Our analysis exhibits inherent limitations due to its observational and non-randomized design. First, frailer patients might not have undergone VTA, and this discrepancy in clinical management could have influenced outcomes in the VTA− arm. While no differences in most baseline characteristics were detected among groups, a significant difference in terms of diabetes was found among them. This discrepancy may have contributed to greater frailty in the VTA− cohort. Second, the choice of ablation over medical therapy was likely associated with operators’ and/or patients’ preferences. Third, the majority of centres involved in this project is third-level referral centres in their country, and therefore, the clinical outcomes of VTA may not be generalizable to less experienced institutions. Fourth, the relatively low number of patients undergoing VTA in our cohort (27.9%) may represent another potential limitation of our analysis. Randomized controlled trials evaluating an invasive strategy vs. medical management in this population are surely needed to confirm our results.

## Conclusions

In a real-world registry of S-ICD carriers, the combined study endpoint of arrhythmic events and cardiovascular mortality was lower in the patient cohort undergoing VTA at long-term follow-up.

## Data Availability

Data supporting this study are available upon reasonable request to the corresponding author.

## Appendix

**List of participating centres and investigators**

**Italy**

Ospedale Luigi Sacco (Polo Universitario), Milan: M. Schiavone, A. Gasperetti, R. Arosio, M. Viecca, G.B. Forleo

Policlinico Sant’Orsola Malpighi, Bologna: M. Ziacchi, I. Diemberger, A. Angeletti, M. Biffi

Ospedale San Raffaele, Milan: N. Fierro, S. Gulletta, P. Della Bella

Centro Cardiologico Monzino, Milan: C. Tondo

Spedali Civili Brescia, Brescia: G. Mitacchione. A. Curnis

Ospedale ‘Umberto I-Salesi-Lancisi’, Ancona: P. Compagnucci, M. Casella, A. Dello Russo

Ospedale G.B. Grassi, Ostia: L. Santini

Ospedale San Filippo Neri, Rome: C. Pignalberi

Policlinico Umberto I, Rome: M. Magnocavallo, A. Piro, C. Lavalle

Campus Biomedico, Rome: F. Picarelli, D. Ricciardi

Policlinico Casilino, Rome: E. Bressi, L. Calò

Ospedale San Gerardo, Monza: E. Montemerlo, G. Rovaris

Ospedale di Castrovillari, Castrovillari: S. De Bonis, A. Bisignani, G. Bisignani

Ospedale Vito Fazzi, Lecce: G. Russo, E. Pisanò

Ospedale Card. Panico, Tricase: P.Palmisano

Ospedale Santa Chiara, Trento: F. Guarracini

AOU Ferrara—Arcispedale S.Anna, Ferrara: F. Vitali, M. Bertini

**Germany**

UKSH Universitätsklinikum Schleswig-Holstein, Lübeck: J. Vogler, T. Fink, R. Tilz

Universitätsklinik Mannheim, Mannheim: F. Fastenrath, J. Kuschyk

Asklepios Klinik St. Georg, Hamburg: L. Kaiser, S. Hakmi

**France**

APHP, Hôpital Pitié Salpêtrière, Paris: M. Laredo, X Waintraub, E. Gandjbakhch, N. Badenco

**Switzerland**

Universitätsspital Zürich, Zurich: A. Breitenstein, A.M. Saguner

**Austria**

Ordensklinikum Linz Elisabethinen, Linz: M. Martine, S. Seidl

**USA**

Montefiore Einstein Center for Heart and Vascular Care, Montefiore Medical Center, Albert Einstein College of Medicine, New York: L. Di Biase
